# Impact of sociodemographic variables on executive functions

**DOI:** 10.1590/1980-57642016dn11-010010

**Published:** 2017

**Authors:** Kenia Repiso Campanholo, Izadora Nogueira Fonte Boa, Flávia Cristina da Silva Araujo Hodroj, Glaucia Rosana Benute Guerra, Eliane Correa Miotto, Mara Cristina Souza de Lucia

**Affiliations:** 1 Division of Psychology, Hospital das Clínicas da Faculdade de Medicina da Universidade de São Paulo, São Paulo SP, Brazil.; 2 Department of Neurology, Hospital das Clínicas da Faculdade de Medicina da Universidade de São Paulo, São Paulo SP, Brazil.

**Keywords:** aging, socioeconomic factors, executive function

## Abstract

Executive functions (EFs) regulate human behavior and allow individuals to interact and act in the world. EFs are sensitive to sociodemographic variables such as age, which promotes their decline, and to others that can exert a neuroprotective effect.

**Objective:**

To assess the predictive role of education, occupation and family income on decline in executive functions among a sample with a wide age range.

**Methods:**

A total of 925 participants aged 18-89 years with 1-28 years' education were submitted to assessment of executive functions using the Card Sorting Test (CST), Phonemic Verbal Fluency (FAS) Task and Semantic Verbal Fluency (SVF) Task. Data on income, occupation and educational level were collected for the sample. The data were analyzed using Linear Regression, as well as Pearson's and Spearman's Correlation.

**Results:**

Age showed a significant negative correlation (p<0.001) with performance on the CST, FAS and SVF, whereas education, income and occupation were positively associated (p<0.001) with the tasks applied. After application of the multivariate linear regression model, a significant positive relationship with the FAS was maintained only for education (p<0.001) and income (p<0.001). The negative relationship of age (p<0.001) and positive relationship of both education (p<0.001) and income (p<0.001and p=0.003) were evident on the CST and SVF.

**Conclusion:**

Educational level and income positively influenced participants' results on executive function tests, attenuating expected decline for age. However, no relationship was found between occupation and the cognitive variables investigated.

## Abstract (pt)

Funções executivas regulam o comportamento humano e permitem ao indivíduo interagir e agir no mundo. Elas são sensíveis a variáveis sociodemográficas como a idade, que promove seu declínio, e a outras que podem ter ação neuroprotetora.

**Objetivo:**

Avaliar o papel preditivo da escolaridade, atividade ocupacional e renda familiar sobre o declínio das funções executivas em uma amostra de ampla variação de idade.

**Métodos:**

925 participantes saudáveis com idades de 18 a 89 anos e escolaridade de 1 a 28 anos foram submetidos ao exame de funções executivas com uma Tarefa de Classificação de Cartas (TCC) e de Fluência Verbal nominal (FVN) e semântica (FVS). Foram obtidas a renda, atividade ocupacional e escolaridade da amostra. Os dados foram analisados com Regressão Linear, Correlação Pearson e Spearman.

**Resultados:**

Idade apresentou correlação significativa (p<0,001) negativa com o desempenho em TCC, FVN e FVS, enquanto educação, renda e ocupação se relacionaram de forma positiva (p<0,001) com as tarefas utilizadas. Após modelo de regressão linear multivariada, apenas educação (p<0,001)e renda (p<0,001) mantiveram relação significativa positiva com FVN. A relação negativa da idade (p<0,001) e positiva de educação (p<0,001) e renda (p<0,001 e p=0,003) foi evidente em TCC e FVS.

**Conclusão:**

Escolaridade e renda influenciaram positivamente os resultados dos participantes nos testes de função executiva, tendo um efeito contrário ao declínio esperado para a idade. Por outro lado a ocupação não manteve relação com as variáveis cognitivas.

## INTRODUCTION

Executive functions (EFs) regulate human behavior and represent a group of abilities that allow individuals to interact and act intentionally in the world, i.e. based on the creation, supervision and readaptation of goals.^1,2^ This involves the formulation of a plan of action and in doing so considers previous individual experiences and the environmental constraints imposed, allowing the devising of an appropriate sequence of actions to execute the initial plan.^1^

The executive functions involve the frontal lobe, particularly the pre-frontal cortex.^3^ The development of EFs commences in the first year of life, and develops most between 6 and 12 years of age.^4^ After complete maturation, the EFs remain stable until the onset of senescence,^3^ being one of the first functions affected by the aging process, particularly working memory^5^ and information processing speed.^6^ With advancing age, hemodynamic changes in the whole brain can be expected, where these are most marked in the frontal, temporal and occipital lobes.^7^ Moreover, age-dependent frontal lobe atrophy,^8^ as well as reduced connectivity between frontal regions and subtentorial territories, has also been described.^8,9^

However, in order to better understand the relationship between advancing age and decline in executive functions, differences in the aging process due to cultural and sociodemographic variability in the population must be elucidated.^10,11^ Exposure to factors such as reading habits,^12^ cognitively-demanding activities,^13^ and especially higher educational levels,^11,12,14-16^ influence base cognitive capacity. These stimuli help maintain effective cognitive performance despite aging or the onset of brain pathologies, thus establishing a neuroprotective relationship.^14,17-21^

Two sociodemographic processes and their influences on cognition are less investigated in the literature, namely, previous occupation and socioeconomic level, where the latter is an item constituting the human development index.^22^

Healthy elderly engaged in life with more complex occupational tasks have better performance on cognitive testing, irrespective of gender, age or educational level.^13^ The hypothesis for these findings is that occupational activities of a high technical level induce mental stimulation through the practice and strengthening of cognitive functions.^13,23^

With regard to the economic aspect, its impact on cognitive development from childhood is clear, since brain development is positively correlated with higher family income.^24^ In adults, greater scores on screening tests correlate with higher incomes.^25^ On a broader level, socioeconomic status is considered indicative of health, and is also associated with a lower tendency for cognitive decline and depression.^26^

However, the cited studies remain limited and are largely based on screening tests. This scenario prompted the present study investigating executive functions sensitive to aging^3^ and their relationships with sociodemographic variables by applying tests sensitive to EFs. Two tasks are often employed in EF assessments. The first is the card sorting test,^27^ which requires recruitment of visual EFs related to categorization, working memory and mental flexibility.^28^ The second is phonemic and semantic category verbal fluency tasks,^29,30^ which require greater recruitment of verbal EFs related to working memory and processing speed,^31^ in addition to language resources such as lexical access.^32^

The role of EFs as regulators of human behavior renders it important to understand how these abilities are maintained during adult life and aging, and particularly the impact of sociodemographic factors on these functions.^21^ Understanding the relationship between these variables and EFs can help promote improvements in quality of life. Therefore, the objective of the present study was to assess the predictive role of sociodemographic variables (educational level, occupation and family income) on EF decline in a sample with a wide age range.

## METHODS

**Participants.** A total of 1,310 Portuguese-speaking natives (Brazil) aged 18-89 years with 1-28 years education were recruited. Individuals with no motor or sensory deficits preventing execution of the tasks, no previous or current history of neurological or psychiatric diseases, and not in use of psychotrophic drugs were initially included.

Of the initial sample, 339 subjects with scores below established cut-off medians for educational level (20 for illiterates; 25 for 1-4 years; 26.5 for 5-8 years; 28 for 9-11 years and 29 for higher education) on the Mini-Mental State Exam (MMSE)^32^ were excluded, comprising 8 for reporting symptoms of anxiety and depression on the Hospital Anxiety and Depression Scale (HADS)^33^ and 38 for having estimated intelligence quotient <80. Thus, 925 participants were included in the study.

**Instruments and procedures.** Education was determined based on the number of years' education counted up until the last full year of school or university attended. Academic years studied due to repeats were not counted.

Occupations were classified according to the International Standard Classification of Occupations (ISCO) into the 10 major groups (0 – armed forces, military police and firemen; 1 – senior officials, legislators, chief executives and managing directors; 2 – science and arts professionals; 3 – technicians and associate professionals; 4 – clerical support workers; 5 – service workers; market and shop salespersons; 6 – Agricultural, forestry, game and fishery workers; 7 and 8 – Trades workers and operators; and 9-maintenance and repair workers.^34^ For the purposes of the present analysis, these 10 major groups were consolidated into 3 categories: General Services (ISCO 4-9), Technical Professionals (ISCO 3) and University Professionals (ISCO 2). The groups ISCO 0 and 1 were distributed amongst these 3 categories according to the educational level required for the job performed. A fourth category was added for this study containing individuals Not in Formal Employment because they were retired, unemployed, students or performing occupational tasks exclusively for their family (homemakers).

Income was calculated based on the number of minimum monthly wages (MWs) received, categorized into five groups: <3 MWs; 4-6 MWs; 7-9 MWs; 10-12 MWs; or ≥13 MWs. The income variable was based on family income (sum of incomes of all dwellers in the same household) as opposed to individual income.

After signing the Free and Informed Consent Form approved by the Ethics Committee of the University of São Paulo School of Medicine (CAPPesq 086/06), the participants were submitted to the semi-structured interview for collection of sociodemographic data and medical history. The inclusion and exclusion criteria were checked by applying the cognitive screening (MMSE) and mood (HADS)^33^ scales, and the assessment of estimated intelligence quotient^35^ based on the Vocabulary and Matrix Reasoning subtests of the Wechsler Adult Intelligence Scale (WAIS-III).^36^

For assessing EFs, two tasks associated with activation of pre-frontal regions were employed.

*Card Sorting Test (CST)(27) –* The participant has to sort 48 cards according to the number, shape and color of their printed stimuli. Feedback was given on each attempt regarding the accuracy of the response. In this study, only the number of complete categories was scored. Performing the task requires the formation of strategies, planning and mental flexibility and thus activates the dorsolateral (DLPFC), ventrolateral (VLPFC) and anterior cingulate (ACC)^37^ cortex regions.

*Phonemic Verbal Fluency (FAS) and Semantic Verbal Fluency (SVF)(30) –* For the FAS, the participant must say as many words as possible in 60 seconds starting with a given letter, abiding by two rules: do not use proper nouns or words with suffixes. The letters used were F, A and S, with score taken as the sum of words produced using the three letters. For the SVF, the participant has to say as many animal names as possible in 60 seconds, with score taken as the sum of the words produced in this category. Letter and semantic category verbal fluency tasks require monitoring of rules and inhibition of inadequate responses, besides language abilities. Therefore, these tasks activate temporal regions and the superior anterior pre-frontal region.^38^ It is important to note that the both the FAS and the SVF directly depend on processing speed, since they are tests which assess the number of responses uttered in a short timeframe, where the examinee must attempt the highest production as possible while abiding by the rules given.^30^

**Statistical analysis.** All statistical analyses were performed using the statistical software SPSS V20 for Windows 8.1. For descriptive statistics, mean was used as the measure of central tendency and standard deviation as the measure of dispersion. Categorical data were expressed as relative and absolute frequency measurements.

Pearson's or Spearman's correlation was used to determine the relationship between the sociodemographic variables and the results on the executive function tests. Variables showing correlation with p≤0.01 were included in the Multivariate Linear Regression Model, with significance set at the level of p≤0.05.

## RESULTS

The socioeconomic and demographic characteristics of the study sample are given in Table 1. Notably, the proportion of participants with very low educational level was small, where 2 participants had 1 year of education, 4 had 2 years and 14 had 3 years' education. Scores by participants on the FAS, SVF and CST are given in Table 2.

**Table 1:** Sample characteristics.

Demographic and cognitive screening variables		Total sample (n=925)
Gender	Male	324 (35%)
	Female	601(65%)
Education	1-8 years(n (%))	306 (33%)
	9-11 years(n (%))	202 (22%)
	>11 years (n (%))	417 (45%)
Age	18-39 years (n (%))	447 (48%)
	40-59 years (n (%))	307 (33%)
	60-69 years (n (%))	120 (13%)
	>69 years (n (%))	51 (6%)
Income	1-3 minimum wages (n (%))	270 (29%)
	4-6 minimum wages (n (%))	239 (26%)
	7-9 minimum wages (n (%))	102 (11%)
	10-12 minimum wages (n (%))	127 (14%)
	>12 minimum wages (n(%))	187 (20%)
Occupation	Not in formal employment (n (%))	237(26%)
	General Services (n (%))	244 (26%)
	Technical-level Professionals (n (%))	107 (12%)
	University-level Professionals (n (%))	337 (36%)
Mood and cognitive screening	Intelligence Quotient (M(SD))	103.50 (12.45)
	Anxiety symptoms (M(SD))	4.45 (2.13)
	Depression symptoms (M(SD))	3.39 (2.29)
	Mini-Mental State Exam (M(SD))	28.99 (1.21)

M: mean; SD: standard deviation.

**Table 2:** Scoring of participants on CST, FAS and SVF tests.

Age		Years' education							
		1 to 8			9 to 11			>11 years	
		N	M (SD)		N	M (SD)		N	M (SD)
18-39 years	FAS Sum of letters	54	28.28 (8.61)		58	31.76 (8.21)		137	41.56 (9.04)
	SVF	54	14.48 (3.05)		58	17.55 (4.49)		137	21.40 (4.60)
	CST	54	3.96 (1.74)		58	4.93 (1.39)		137	5.67 (0.79)
40-59 years	FAS Sum of letters	72	31.93 (9.41)		49	32.33 (6.85)		77	43.06 (10.63)
	SVF	72	14.58 (3.48)		49	15.86 (4.10)		77	20.43 (4.06)
	CST	72	4.15 (1.81)		49	4.78 (1.56)		77	5.48 (1.26)
60-69 years	FAS Sum of letters	47	27.00 (7.81)		41	34.22 (8.11)		83	40.31(10.55)
	SVF	47	12.89 (3.66)		41	15.98 (3.47)		83	18.63 (4.18)
	CST	47	4.40 (1.51)		41	5.05 (1.27)		83	5.52 (0.88)
70-89 years	FAS Sum of letters	34	32.21 (7.45)		31	33.65 (7.81)		71	39.66 (9.57)
	SVF	34	14.03 (2.44)		31	15.00 (3.77)		71	19.18 (5.57)
	CST	34	4.56 (1.71)		31	4.13 (1.43)		71	5.32 (1.16)

CST: Card Sorting Test; FAS: Phonemic Verbal Fluency Test; SVF: Semantic Verbal Fluency Test; M: mean; SD: standard deviation.

The correlations between the sociodemographic variables and EF tests are given in Table 3. The results of these analyses revealed a negative relationship between age and the cognitive variables, suggesting poorer results on the tests applied with increasing age. A positive relationship was found between the cognitive variables and the measurements of income, educational level and occupation, suggesting that an increase in one of these variables was concomitant with increase in the others.

**Table 3:** Correlation between executive functions and sociodemographic variables.

		Age**	Years' education**	Occupation^†^	Income^†^
FAS Sum of letters	r	-0.113*	0.576*	0.410*	0.410*
	p	<0.001	<0.001	<0.001	<0.001
SVF	r	-0.268*	0.553*	0.446*	0.372*
	p	<0.001	<0.001	<0.001	<0.001
CST	r	-0.162*	0.417*	0.320*	0.376*
	p	<0.001	<0.001	<0.001	<0.001

* Significant correlation p<0.01;

** Pearson's correlation;

† Spearman's correlation. CST: Card Sorting Test; FAS: Phonemic Verbal Fluency Test; SVF: Semantic Verbal Fluency Test.

Based on these results, a multiple linear regression model was constructed to assess the effects of education, income and occupation on the scores for the EF tasks. The results of the analysis are given in Table 4.

**Table 4:** Multiple linear regression model of performance on executive functions tasks by age, education, occupation and income.

Variables		B	SE (B)	β	p	R^2^
FAS Sum of letters	Age	-0.015	0.020	-0.023	0.436	0.342
	Years' education	0.946	0.079	0.469	<0.001**	
	Occupation	0.366	0.329	0.042	0.267	
	Income	0.914	0.234	0.131	<0.001**	
SVF	Age	-0.057	0.009	-0.181	<0.001**	0.341
	Years' education	0.414	0.037	0.435	<0.001**	
	Occupation	0.184	0.156	0.045	0.238	
	Income	0.324	0.111	0.099	0.003**	
CST	Age	-0.012	0.003	-0.130	<0.001**	0.208
	Years' education	0.081	0.012	0.281	<0.001**	
	Occupation	-0.014	0.051	-0.012	0.784	
	Income	0.210	0.037	0.212	<0.001**	

** Significant correlation p<0.01. CST: Card Sorting Test; FAS: Phonemic Verbal Fluency Test; SVF: Semantic Verbal Fluency Test.

The analysis revealed that the significant positive relationship of education and income with the FAS Sum of Letters remained. Income and education explained 34% of the results on the FAS Sum of Letters.

The positive relationship of education and income and SVF was maintained, whereas age retained its negative relationship despite having a lesser impact on this cognitive variable than the other factors. Age, education and income explained 34% of the results on the SVF.

Lastly, for the CST, the negative relationship with age remained, whereas age and income were positively associated with the cognitive variable. The relationship with income was the strongest. Age, education and income explained 21% of the results on the CST.

Notably, the positive relationship of occupation with the EF variables did not persist on the linear regression model.

## DISCUSSION

The objective of the present study was to investigate the predictive value of sociodemographic variables (educational level, occupation and family income) on decline in executive functions. In order to better understand this relationship, the impact of cultural and sociodemographic population variability on age-related cognitive performance must be elucidated.^10,11^

The distribution of gender, age and education of the study sample mirrored that of the general Brazilian population^39^ and also the pattern reported in other Brazilian studies.^13^ Thus, the present sample is sociodemographically similar to the latest 2016 report by the Brazilian Institute of Geography and Statistics (IBGE). The findings of this study revealed that increased age is associated with lower scores on the executive function tests. However, the relationships between sociodemographic status and scores on the same tasks were positive, indicating that better social (occupation and income) and educational status correlate with better performance on the tasks.

Similar studies have shown that performance on cognitive tasks is more strongly associated with greater occupational complexity than other variables such as education.^13,23^ However, the cited studies employed other assessment instruments without specific focus on EFs. Also with regard to income, the accumulation of wealth during life indicates better social and health status^40,41^ and can therefore be considered an indicator of health aging.

Concerning the impact of education, its neuroprotective effect on cognitive decline is supported by the literature.^11,12,14-16,42^ Studies show that access to higher educational levels is associated with greater scores by elderly on cognitive tasks^12,15,42^ involving EFs and other domains^5,6,43,44^

However, in order to determine the mutual relationship of these variables with EFs, linear regression analysis was conducted. The results showed that increased age maintained a negative effect on SVC and CST scores, whilst education and income retained a positive relationship. The negative relationship of age with the FAS test no longer remained, whereas the positive relationship of income and education was maintained, serving as predictors of better results independently of age. Occupation, despite exhibiting a positive relationship with the EF variables when analyzed alone, did not retain this relationship after inclusion in the regression analysis models.

However, it should be noted that the information on income in this study was not based on the participate alone but on the family. Hence, the participant may not be engaged in paid work, yet report a high income due to the occupations of family members. Higher family income, in turn, is indicative of greater access to education.^45^ These facts can explain the predictive values of better results on EF tests in groups with higher income and education.

The results also showed that the FAS was not influenced by age whereas SVF performance correlated negatively with age. A Portuguese language study showed similar results,^46^ perhaps explained by the sensitivity of the SVF to activity decline in brain regions with increasing age.^46^ The phonemic verbal fluency task requires literacy, being more strongly associated with educational level and involving other brain regions that provide support in aging.^45,47^

Measures of information processing speed and working memory can shed further light on the findings of this study, given these measures are sensitive to socioeconomic variables and may have a relationship with the results outlined. Therefore, it is envisaged that future studies can contribute further.

Finally, the results of the present study revealed that educational level and income positively influenced participants' results on EF tests, attenuating expected decline for age. However, the relationship of occupation with the cognitive variables did not persist after inclusion in the linear regression model.
